# Depression, anxiety and related factors among Syrian breast cancer patients: a cross-sectional study

**DOI:** 10.1186/s12888-022-04469-y

**Published:** 2022-12-17

**Authors:** Jameel Soqia, Mohammed Al-shafie, Laila Yakoub Agha, Mhd Basheer Alameer, Dima Alhomsi, Rakan Saadoun, Maher Saifo

**Affiliations:** 1 Faculty of Medicine, Damascus University, Damascus, Syria; 2 Medical Faculty Mannheim, Ruprecht Karls University Heidelberg, Mannheim, Germany; 3 Department of Otorhinolaryngology-Head and Neck Surgery, Mannheim Medical Center, Mannheim, Germany; 4 Department of Plastic Surgery, The University of Pittsburgh, Pittsburgh, USA

**Keywords:** Depression, Anxiety, Breast cancer, PHQ, GAD, Syria

## Abstract

**Introduction:**

Breast cancer represents a traumatic experience with a psychological burden. The prevalence of psychological distress (which include depression and anxiety) among breast cancer patients is estimated to be 15 to 54%, but studies have shown that applying some psychological treatments has contributed to decreasing depression and anxiety. So, it is crucial to diagnose and treat patients with the appropriate means. After reviewing the literature, no studies discussed depression and anxiety among Syrian breast cancer patients.

**Methods:**

A cross-sectional study in Al-Bairouni hospital in Damascus, Syria carried out using face-to-face interviews based on a structured questionnaire. All breast cancer patients were included, except who refused to participate, and responses with missing data were excluded. The questionnaire consisted of 2 sections: the first included Socio-demographic characteristics, and the other evaluated patients' depression using PHQ-2 and GAD-2 scales. Data were gathered using the Kobo toolbox app and then entered into an Excel sheet.

**Results:**

Five hundred patients were interviewed. 35.6% of the patients had a GAD-2 score greater than or equal to 3.00, while 35% had a PHQ-2 score greater than or equal to 3.00. There is a significant negative relationship between the age of the patient and the GAD-2 score and PHQ-2 score, which means the older the patient is, the lower the GAD-2 and PHQ-2 scores are. A multivariable regression model showed that younger (age ≤ 45 years) and being widowed were associated with being positive for further evaluation for generalized anxiety disorder. Similarly, patients younger than 45 are significantly associated with the need for further evaluation for major depressive disorder (MDD). Social status had a stronger association with the need for further assessment for MDD, with divorced women showing the strongest association, followed by widowed and married women compared to single women.

**Conclusion:**

This study showed high anxiety and depression among breast cancer patients. The patient’s age and social status were significant factors in determining the need for further psychological assessment. In General, Younger patients showed higher levels of depression and anxiety, the size of the tumor did not show significant association with psychological distress.

## Introduction

Breast cancer is the most common cancer in humans, with 7.8 million cases reported in the last five years [1]. Breast cancer incidence rate has risen in the Arab world between 1990 and 2016, with expectations of increasing even more in the following ten years [2]. Besides physical manifestations, breast cancer causes a psychological burden on patients [3, 4], caused by the illness itself or the treatment prescribed to them, which has been described as a traumatic experience [5]. This psychological burden may present as anxiety, depression, anger, uncertainty about the future, hopelessness, desperateness, fear of recurrence of cancer, fear of separation from relatives, fear of pain, decrease in self-esteem, impairment of body image, the anxiety of not being loved or shown interest, and fear of death [5]. Moreover, psychological distress (which include symptoms of depression, anxiety or stress) is connected with suffering from clinically significant pain [6], and patients are more likely to have suicidal thoughts [7]. The prevalence of psychological distress is estimated at around 35% in cancer patients [8], while it is estimated to be 15 to 54% in breast cancer patients [9]. Many factors affect the prevalence of psychological distress in breast cancer patients (age, marital state, education, employment, cancer at a young age) [10]. Implementing a form of psychological therapy has shown a decrease in psychological symptoms [11] and a better adherence to treatment [5]. Arab societies usually think of unavoidable death when discussing cancer [12] which affects their mental health. Furthermore, cancer patients suffer from the societal stigma imposed on cancer patients [13, 14]. Religion and family are major factors affecting the coping of cancer patients in the Arab world serving as a coping mechanism and a source of increased stress [13, 14]. Due to the above evaluating psychological disorders in breast cancer patients is a must to detect and treat patients with the appropriate means such as active listening, emotional reassurance, psychological counseling, and pharmacotherapy [15] to ensure a better quality of life [16]. After reviewing the literature, no studies discussed psychological disorders in Syrian breast cancer patients on a large scale. However, this kind of data is important to draw attention needed to highlight this problem. Which is the first step to solving it,for instance, by implementing some measures such as training health care providers to screen and treat psychologically affected patients, as well as offering psychological counseling within the hospital, and form an understanding of how the Syrian crisis affected cancer patients psychologically via future research. This study was conducted et al.-Bairouni university hospital in Damascus – Syria, the only center specializing in treating cancer patients in the country [17] which can be a source of stress to patients living in geographically distant areas of the country, to evaluate the prevalence of two psychological distress-related disorders; depression and anxiety in Syrian breast cancer patients and the factors affecting it.

## Methods

### Participants and sampling

This cross-sectional study included Syrian women diagnosed with breast cancer who visited Al-Bairouni Hospital in Damascus, Syria. During the investigation, 500 breast cancer patients were chosen among patients attending the hospital to administer anti-cancer drugs. The whole population of patients who attended the hospital during the time from 15/10/2021 to 16/11/2021 when we conducted our study was 560. Sixty patients refused to participate in the study.

### Data collection

The research team collected data using a structured questionnaire administered via face-to-face interviews with patients in drug administrating rooms. We obtained participants' verbal consent after ensuring to maintain the secrecy of information, each interview lasted approximately 7–10 min. All women diagnosed with breast cancer were included, whereas women who refused to participate, and responses with missing data were excluded.

### Questionnaire

We adopted our questionnaire from surveys in similar previous studies [18] as a point of reference for validation. The questionnaire consisted of 2 sections:

- Section 1 included socio-demographic characteristics (Age, Marital status, Occupation, Education, Governorate of origin, and Family history of breast cancer).
- Section 2 evaluated patients' depression using the Patient Health Questionnaire-2 (PHQ-2) and the Generalized Anxiety Disorder-2 (GAD-2) scales. PHQ-2 and GAD-2 questionnaires are considered brief instruments to measure depression and anxiety. Their validity was evaluated in previous studies [19, 20]. Each questionnaire consists of two questions, and patients should choose their answer from a four-point scale ranging from "0 = not at all" to "3 = nearly every day". And then, we estimate the total scores of PHQ-2 and GAD-2 by summing the scores of the two questions in each questionnaire, getting a range from 0 to 6, with a higher score indicating worse mental health. A score of ≥ 3 in PHQ-2 and GAD-2 suggests the need for further evaluation for major depressive disorder or anxiety disorder [21].

We used the Arabic version of these questionnaires, which were previously validated [22]. Additionally, we assessed their reliability using Cronbach's alpha coefficient, which showed an acceptable value of 0.98 for GAD-2 and 0.90 for PHQ-2.

### Ethical consideration

The study complies with the Declaration of Helsinki for research involving human subjects. The Ethical Committee approved this study in the Faculty of Medicine at Damascus University, Syria (3835, 12–6-2022). All our methods were carried out following relevant guidelines and regulations. Informed consent was obtained from all the participants included in the study. We explained the purpose of the survey to each participant and the way to answer the questionnaire; participation was voluntary. No names were taken, so we provided anonymous data collection.

### Statistical analysis

We gathered data using the Kobo toolbox app and used SPSS version 25 for data analysis. Demographics and clinical characteristics were described using means and standard deviations for continuous variables and by outcome status using frequency and percentage for categorical variables. Independent t-test and One-way ANOVA were used to compare means of continuous variables. The Chi-square test was used to compare the occurrence rates between categorical variables. Pearson correlations were performed to analyse the relationships between variables. A multivariate regression model was fitted to test the adjusted association between the need for further evaluation for depression or anxiety while controlling for other significant variables. The model was built using the enter method. The variables entered in the model were found to have a significant association with the need for further evaluation for MDD or GAD during the initial unadjusted analysis. A *P*-value of < 0.05 was considered statistically significant.

## Results

A total number of 500 patients were interviewed. 310 (62%) of patients were older than 45 years, whereas 188 (37.6%) were between 25 and 45 years old, and only 2 (0.4%) were less than 25 years old. Also, 14.8% of patients had a first-degree relative diagnosed with breast cancer, while 23.6% had a second-degree relative diagnosed with breast cancer. Other sample details and characteristics are shown in Table 1.

**Table 1:** Characteristics of the sample and rate of screening mandatory as a function of baseline demographic and clinical features

Variable	Total (*n* = 500)	GAD-2 Mean (SD)	*P*-Value	PHQ-2 Mean (SD)	*P*-Value	GAD-2 ≥ 3 (*n* = 178)		PHQ-2 ≥ 3 (175)	
	No. (%)					No. (%)	*P*-value	No. (%)	*P*-value
**Age range**									
≤ 45	190 (38.0)	2.6 (2.3)	0.016	2.32 (2.3)	0.018	80 (16.0)	0.017	76 (15.2)	0.066
> 45	310 (62.0)	2.1 (2.3)		1.8 (2.3)		98 (19.6)		99 (19.8)	
**Social Status**									
Single	52 (10.0)	1.6 (2.1)	0.060	1.5 (2.0)	0.059	13 (2.6)	0.193	10 (2.0)	0.026
Married	402 (80.0)	2.3 (2.3)		2.0 (2.9)		144 (28.8)		144 (28.8)	
Divorced	12 (2.0)	3.3 (2.6)		3.3 (2.2)		6 (1.2)		7 (1.4)	
Widowed	34 (7.0)	2.7 (2.4)		2.4 (2.7)		15 (3.0)		14 (2.8)	
**Level of education**									
Illiterate	83 (17.0)	2.2 (2.3)	0.350	1.9 (2.2)	0.430	26 (5.2)	0.488	26 (5.2)	0.333
School^a^	304 (61.0)	2.4 (2.4)		2.1 (2.4)		113 (22.7)		115 (23.1)	
Some university courses	20 (4.0)	2.7 (2.6)		1.8 (2.3)		9 (1.8)		7 (1.4)	
University degree	92 (18.0)	1.9 (2.3)		1.7 (2.2)		29 (5.8)		26 (5.2)	
**Employment’s status**									
Housewife	380 (76.0)	2.4 (2.3)	0.086	2.1 (2.3)	0.206	140 (28.1)	0.329	139 (27.9)	0.206
Employed	119 (24.0)	2.0 (2.3)		1.8 (2.2)		38 (7.6)		36 (7.2)	
**1**^**st**^** degree relatives’ family history of breast cancer**									
No	426 (85.0)	2.3 (2.4)	0.179	2.0 (2.3)	0.368	156 (31.2)	0.253	150 (30.0)	0.812
Yes	74 (15.0)	1.9 (2.3)		1.8 (2.3)		22 (4.4)		25 (5.0)	
**2**^**nd**^** degree relatives’ family history of breast cancer**									
No	382 (76.0)	2.3 (2.4)	0.432	2.0 (2.3)	0.759	138 (27.6)	0.658	133 (26.6)	0.877
Yes	118 (24.0)	2.1 (2.2)		1.9 (2.2)		40 (8.0)		42 (8.4)	

Values are rounded to the nearest 0.1% or the tent place

^a^Elementary, primary, or secondary

The mean total score of the GAD-2 and PHQ-2 questionnaires were 2.28 (SD = 2.34) and 2.00 (SD = 2.30), respectively. 35.6% of patients (178/500) had a GAD-2 score greater than or equal to 3.00 (≥ 3), while 35% of patients (175/500) had a PHQ-2 score greater than or equal to 3.00 (≥ 3). Furthermore, there is a significant negative relationship between the age of the patient and the GAD-2 score r (498) = -0.113, *p* = 0.012, which means the higher the age is, the lower the GAD-2 score is (and the opposite is true). Also, there is a significant positive relationship between the PHQ-2 and GAD-2 score r (498) = 0.737, *p* < 0.001.

Divorced Patients (*n* = 12) had the highest GAD-2 score, a mean of 3.33. Widows (*n* = 34) had the second-highest GAD-2 score, with a mean of 2,67. While, married patients (*n* = 402) came at the third-highest GAD-2 score, mean of 2.30. Finally, single participants (*n* = 52) had the lowest GAD-2 score, a mean of 1.63.

Work status nor education level, the governorate of origin, or family breast cancer history significantly had a difference on GAD-2 score, with a p-value of 0.230, 0.223, 0.199, and 0.180, respectively.

Additionally, there is a significant negative relationship between the patient's age and PHQ-2 score r (498) = -0.107, *p* = 0.017. Divorced Patients (*n* = 12) had the highest PHQ-2 score, a mean of 3.33. In comparison, widows (*n* = 34) had the second-highest PHQ-2 score, a mean of 2.38. Furthermore, married Patients (*n* = 402) came at the third-highest PHQ-2 score, mean of 2.00. Finally, single patients (*n* = 52) had the lowest PHQ-2 score, a mean of 1.50. Moreover, neither work status nor social status, education level or governate (the place of living) or family breast cancer history significantly impacted PHQ-2 score, with a p-value of 0.449, 0.059, 0.370, 0.173 and 0.369, respectively.

In a multivariable regression model for the risk of being in need for further evaluation for general anxiety disorder (GAD) that counts for the age range and for social status, younger (age ≤ 45) and being widowed were associated with being positive for further evaluation for generalized anxiety disorder, (OR, 1.646, 95%CI: 1.125–2.408) and (OR, 2.742, 95%CI:1.075–6.993), compared to those younger than 46 and single patients respectively (Table 2).

**Table 2:** Multivariable logistic regression analysis for screening mandatory based on the GAD-2 and PHQ-2

Variable	Adjusted odds ratio (95% CI)
**GAD-2 score ≥ 3**	
Age	
Age ≤ 45	1.646 (1.125–2.408)
Age > 45	**Reference**
Social status	
Single	**Reference**
Married	1.732 (0.891–3.368)
Divorced	3.086 (0.838–11.368)
Widowed	2.742 (1.075–6.993)
**PHQ-2 ≥ 3**	
Age	
Age ≤ 45	1.485 (1.012–2.179)
Age > 45	**Reference**
Social status	
Single	**Reference**
Married	2.411 (1.171–4.965)
Divorced	6.031 (1.1751–23.151)
Widowed	3.300 (1.237–8.803)

Similarly, younger patients had a significant association with the need for further evaluation for major depressive disorder (MDD) in a multivariable regression model that counts for social status (OR, 1.485, 95%CI: 1.012–2.179). However, in this model, social status had a stronger association with the need for further evaluation for MDD, with divorced women showing the strongest association (OR 6.031 95%CI: 1.1751–23.151), followed by widowed (OR 3.300 95%CI: 1.237–8.803) and lastly married women (OR: 2.411, 95%CI: 1.171–4.965) when compared to single women.

## Discussion

Anxiety is our body’s natural response to stressful, dangerous, and unfamiliar situations, while depression is the feeling of sadness and a lack of interest in previously enjoyable activities [23]. Both conditions can highly affect people's lives, causing negative changes to their functionality and productivity. Getting breast cancer is not an easy situation and is considered a life-changer and a great cause of anxiety and major depression for patients. The treatment of cancer must include all human aspects, including the psychological one, as a previous study had proven the benefit of psychological treatment in increasing the survival rate of breast cancer patients [24, 25].

Depending on the GAD-2 scale less than 40% of the patients needed further evaluation for GAD [26]. This finding goes in line with a study published by Tsaras, K., et al. among breast cancer patients in Greece [18].

Patients with a PHQ-2 score of 3 or higher are more likely to suffer from MDD [27]. 35% of our patients had a PHQ-2 score ≥ 3. These results are similar to a previous study from Greece [18] and comparable to the findings of previous literature [28, 29]. Most of the published studies reported more elevated symptoms of anxiety than depression [18, 30]. In our work, the percentages of patients who are expected to suffer from GAD or MDD were similar. This is probably due to the cumulative effect of the risk factors and stressors that Syrians have been recently experiencing, including bad financial situation, loss of a loved one, and poor life circumstances. All of these may have contributed to the increase in the risk of MDD [31, 32].

Our results showed that the age of the patient and their social status had a significant effect on the probability of the need for further psychiatric evaluation; the younger the patient is the higher possibility of her suffering from anxiety and depression. This is similar to the finding of a previous study conducted in the USA [33] and a study conducted in the American University in Beirut [34]. Younger females with breast cancer are more concerned about their loss of attractiveness and femininity, especially if the treatment includes breast removal, and constant worrying about breastfeeding after breast cancer treatment.

Published studies stressed the role of support by family and beloved ones surrounding the patients in decreasing the risk of anxiety and depression [35, 36]. This was evident also in our population. In the screening for MDD, social status had a stronger association than age with the need for further evaluation. Even though single, divorced, and widowed women technically have no partner to rely on, the Divorced and widowed may have more burdens and responsibilities toward their children compared to single women, who are usually childless in Syria and live with their families. This result goes in line with a previously published study by G. Bulotiene., et al. among 117 breast cancer patients [37]. In a large retrospective review with over 35,000 patients, Ding, Wu et al. found that married women had better survival and stressed the need to provide patients without a partner with suitable social support. The presence of a partner who could have helped in housekeeping and care for the children may play a role in supporting the patient's psychological health [38]. In GAD-2, widowed women have an elevated association with the need for further evaluation for GAD; they are usually the head of the household with children, and the idea of leaving those children without support may be a significant stressor for them.

This study showed that the level of education did not significantly affect the patient's psychological status. Educated women might be more aware of cancer as well as be more able to gain the information needed to cope with their condition and be more satisfied with the treatment [39]. On the other hand, educated patients might feel overstressed and start to expect the worst the more they know about their cancer and use rumination and self-criticism to cope with cancer resulting in anxiety and depression [40, 41].

We did not find any impact of the occupation on patients' level of anxiety and depression; This finding contradicts previous literature[38, 42–44]. Ding, W., et al. found that working may negatively affect patients' psychological status. As having a demanding job may result in stressing the patient due to her inability to keep up with work and lack of focus as well as job characteristics which can make the female lose her self-confidence and worth [34]. Conversely, working might be a distraction from cancer, providing a supportive community for the patient and a source to keep feeling worthy [42–44]. Nevertheless, work did not affect breast cancer patients included in our study, this can be since most of the women in our study were housewives, had a comfortable job with a flexible schedule or took a break from work, and some of them might be forced to quit working to rest since Arabian families do not count on the woman for financial support. Surprisingly, governorate did not have any effect on the psychological health of the breast cancer patients. In contrast, a previous study showed that greater anxiety and depressive symptoms were mostly documented among cancer survivors in the rural areas as compared to nonrural areas [45].

Having a family history of breast cancer and the level of anxiety or depression were not correlated. This is contradictory to a previous study by Yu Liu et.al. that demonstrated that patients with positive family history showed much stronger anxiety and depression [46].

Larger tumor size may indicate a more progressive stage of the cancer which requires more aggressive treatment methods resulting in the exhaustion of the patient mentally and physically [47]. Therefore, the effect of the tumor size on the level of anxiety and depression of the patient was tested. We collected information about the tumor size of only 165 patients out of 500. Surprisingly, tumor size did not have any effect on the psychological status of the patient. The percentages of the patients whose GAD and PHQ scores were greater or equal to 3 are almost the same in patients with different tumor sizes (average of 33% and 32% in order), which means that tumor size was not an important factor in increasing the risk of the patient's anxiety and depression. The previous statement could be explained by the fact that most of the patients (when interviewed) reported that they did not know their tumor size.

### Limitation

The study was conducted in only one therapy center (Al-Bairouni hospital), so the sample cannot be considered representative of the Syrian community; further studies must include multiple breast cancer therapy centers. Additionally, no information about the staging of the disease or the treatment (The surgical technique, radiotherapy or chemotherapy) were available to discuss in this study.

### Recommendations

Given the results of this study, we recommend that health care providers pay more attention to the social support and psychological aspect of those patients. Screening for depression should be integrated into their follow-up routine to determine their need for psychiatric interventions and improve their quality of life. A specialized psychiatric clinic should be established to assure providing this psychological care. Careful screening for MDD should be awarded for young women who lost their partner for any reason, and careful screening for GAD should be awarded to widowed women. Their family and beloved ones should be counseled about providing support after the patient consent to do so.

## Conclusion

In conclusion, this study showed high anxiety and depression among breast cancer patients. The patient’s age and the social status were major factors influencing her psychological status; younger patients showed higher levels of depression and anxiety, and widowed, divorced, and single women had higher levels of anxiety and depression than married women. There is obvious neglect of the psychological aspect in Syria and a lack of psychological support for breast cancer patients.

## Data Availability

The datasets generated and analyzed during the current study are not publicly available to protect participants’ privacy but are available from the corresponding author on reasonable request.

## Abbreviations

PHQ: Patient health questionnaire
GAD: Generalized anxiety disorder
ANOVA: Analysis of variance
USA: United States of America
